# Dataset of *Arabidopsis* plants that overexpress *FT* driven by a meristem-specific KNAT1 promoter

**DOI:** 10.1016/j.dib.2016.06.002

**Published:** 2016-06-08

**Authors:** L. Duplat-Bermúdez, R. Ruiz-Medrano, D. Landsman, L. Mariño-Ramírez, B. Xoconostle-Cázares

**Affiliations:** a Departamento de Biotecnología y Bioingeniería; Centro de Investigación y de Estudios Avanzados del Instituto Politécnico Nacional. Av. IPN 2508 San Pedro Zacatenco, 07360 Mexico City, Mexico; b Computational Biology Branch, National Center for Biotechnology Information, National Library of Medicine, National Institutes of Health, United States of America, Bethesda, MD, USA

**Keywords:** Differential expression, Bioinformatics, Flowering

## Abstract

In this dataset we integrated figures comparing leaf number and rosette diameter in three *Arabidopsis FT* overexpressor lines (AtFTOE) driven by KNAT1 promoter, “A member of the KNOTTED class of homeodomain proteins encoded by the STM gene of *Arabidopsis*” [5], vs Wild Type (WT) *Arabidopsis* plats. Also, presented in the tables are some transcriptomic data obtained by RNA-seq Illumina HiSeq from rosette leaves of *Arabidopsis* plants of AtFTOE 2.1 line vs WT with accession numbers SRR2094583 and SRR2094587 for AtFTOE replicates 1–3 and AtWT for control replicates 1–2 respectively. Raw data of paired-end sequences are located in the public repository of the National Center for Biotechnology Information of the National Library of Medicine, National Institutes of Health, United States of America, Bethesda, MD, USA as Sequence Read Archive (SRA). Performed analyses of differential expression genes are visualized by Mapman and presented in figures. “Transcriptomic analysis of *Arabidopsis* overexpressing flowering locus T driven by a meristem-specific promoter that induces early flowering” [2], described the interpretation and discussion of the obtained data.

**Specifications Table**

**Table:** 

Subject area	Biology
More specific subject area	Plant Sciences
Type of data	Figure; tables
How data was acquired	PCR final point and ddPCR (The QX100 Droplet Digital PCR (ddPCR™ System), direct count of leaves and measurement of rosette diameter with image analysis software (Imagej [http://rsb.info.nih.gov]), RNA-seq by Illumina HiSeq Sequencing,
Data format	Analyzed
Experimental factors	Three lines overexpressing *FT* (AtFTOE) and WT *Arabidopsis* plants
Experimental features	Three lines of AtFTOE and WT *Arabidopsis* plants were grown on hydroponic under controlled conditions at 22 °C in short day (SD) photoperiod (8 h light /16 h dark) up to day 21 and then transferred to inductive conditions of long days (LD) photoperiod (16 h light /8 h dark)
Data source location	Mexico City, Mexico and at the National Center for Biotechnology Information (NCBI)
Data accessibility	Data is available with this article and at NCBI accession numbers SRR2094583 and SRR2094587

**Value of the data**

• The amplification of transgene by PCR and copy variation number by ddPCR is fundamental to compare independent transgenic events.
• ANOVA and *T*-Student test were employed to assess statistical significance of data (*α*=0.05) for leaf count and rosette diameter in order to compare three AtFTOE lines and WT.
• Differentially expressed genes in the context of cellular functions are graphically presented by Mapman to understand the integrative changes in the metabolism.
• Raw data from the Illumina HiSeq sequencing are available for further analyses.

## 1 Data

In this article are presented the data analyses (figures) from leaf count and rosette diameter for three lines AtFTOE (2.1, 3.1 an 4.3) compared with WT *Arabidopsis* plants (Fig. 2A and B respectively). Data corresponding to differential expression (log2 fold change) from AtFTOE 2.1 line vs WT *Arabidopsis* are visualized by Mapman (Fig. 3). Some data corresponding to down-regulated genes are presented in Table 3.

## 2 Experimental design, materials and methods

### 2.1 Determination of transgene (*FT*) and copy variation number in three AtFTOE lines

**Fig. 1:**
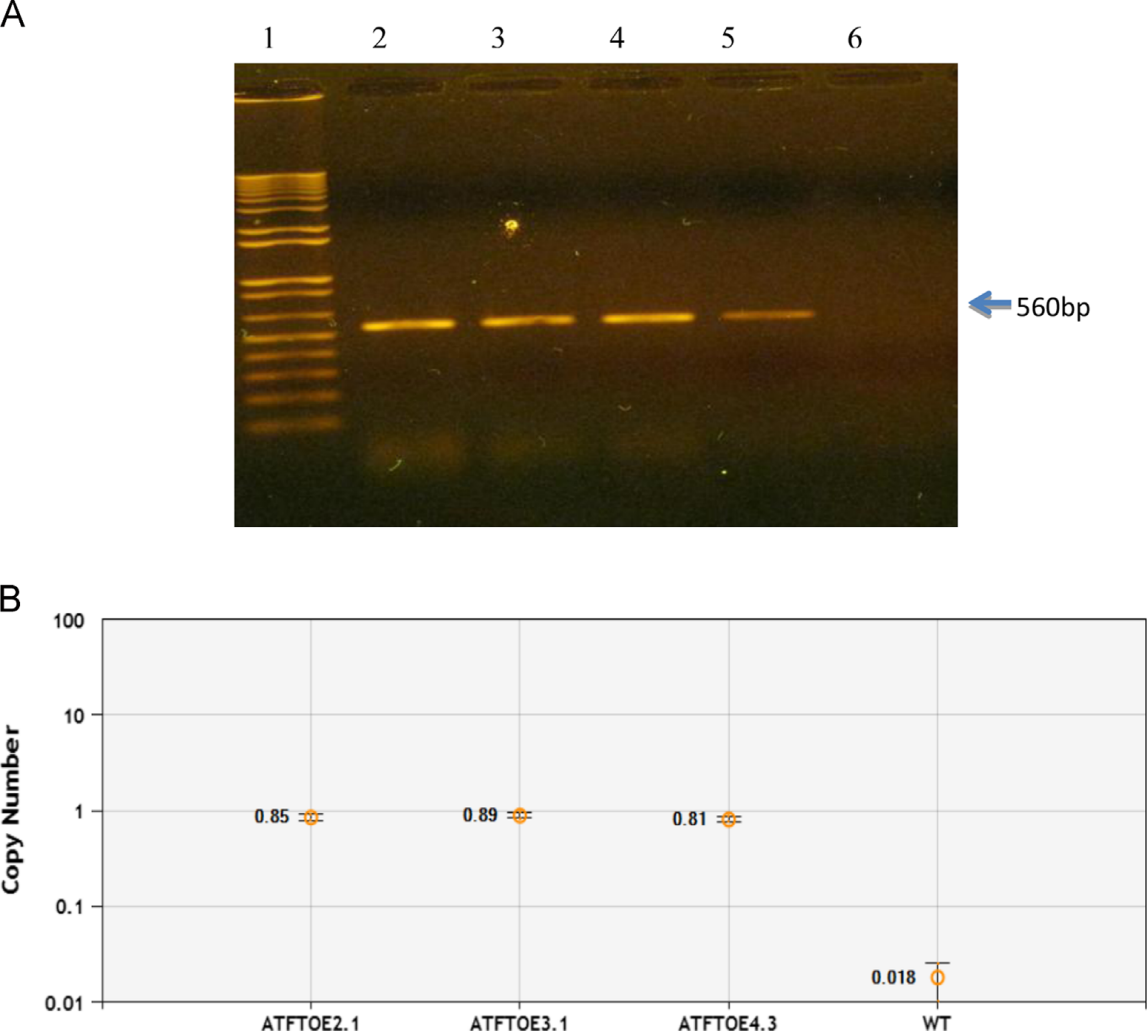
Amplification of transgene *FT* and copy number Variation in three AtFTOE lines (A) 560 bp amplified fragment correspondent to the size of the *FT* transgene visualized in 0.8% agarose gel stained with ethidium bromide. Carril 1: 1 kb plus DNA ladder (Thermo Fisher Scientific®); Carril 2: AtFTOE 2.1; Carril 3: AtFTOE 3.1; Carril 4: AtFTOE 4.3; Carril 5: WT Copy variation number of three AtFTOE lines determined by dPCR™ System (The QX100 Droplet Digital PCR) (B) Copy variation number of three AtFTOE lines determined by dPCR™ System (The QX100 Droplet Digital PCR).

Transgene *FT* (560 bp) was amplified by PCR from three AtFTOE (2.1,3.1 and 4.3) lines (Fig. 1A). Droplet digital PCR (ddPCR) was employed to determine transgene (*FT*) copy variation number (CVN) (Fig. 1B). As template were used 2.5 ng of genomic DNA previously digested with HindIII. Droplets were generated for PCR reaction with the specific primers AtFT-qPCR (F) (5′-TCCGTTTAATAGATCAATCAC-3′), FT-qPCR (R) (5′-CCACCATAACCAAAGTATAG-3) and probe TaqMan ddPCRFT [5′FAM] TCCTGAGGTCTTCTCCACCA [3′BHQ1]. The 152 bp of PCR-amplified product *Arabidopsis HMGB1* (AT3G51880) was used as an internal, reference gene. The primers used as reference were HMGB1 probe [5′HEX]AGGCACCGGCTGAGAAGCCT[3′BHQ1], HMGB1F (5′-CAGAAAGGTGGGAAAGAGGA-3′) and HMGB1-R (5′-AAGGACCCAAACAAACCAAA-3′). The HMGB1 PCR-amplified product was 96 bp. After cycling, the PCR nano droplets were counted using the droplet reader Bio-Rad QX100 system (Bio-Rad 2012).

### 2.2 Determination of leaf number and rosette diameter in three AtFTOE and WT plants

**Fig. 2:**
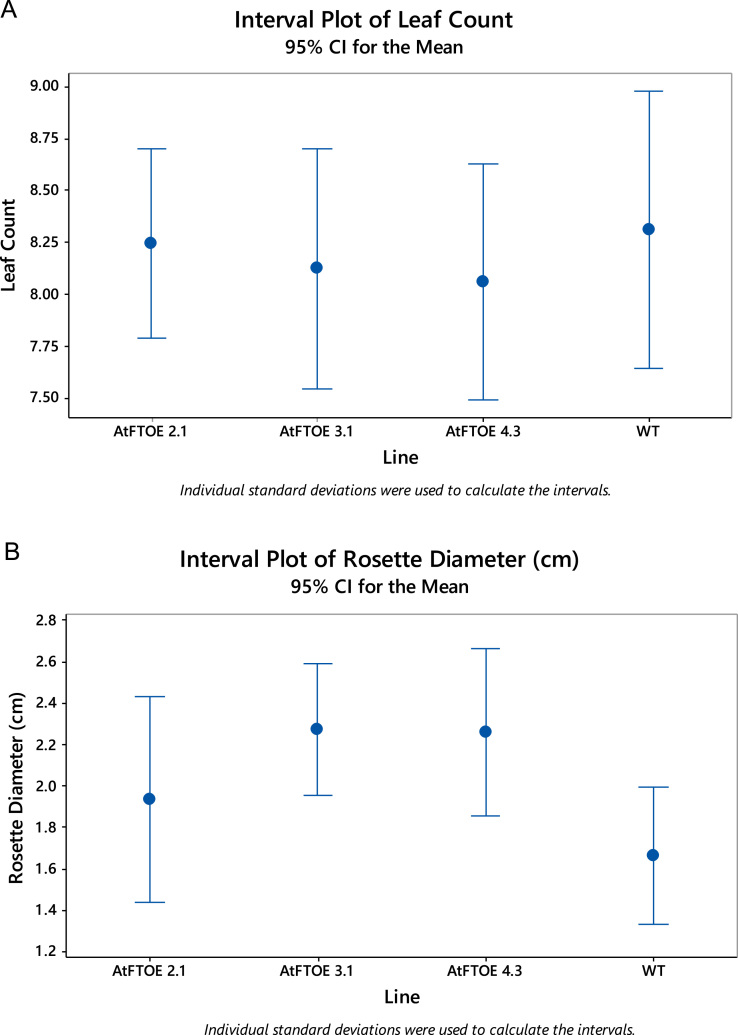
Interval plot from AtFTOE lines and WT for (A) Leaf count and (B) Rosette diameter at 21 days in SD conditions.

Three AtFTOE (2.1,3.1 and 4.3) lines and WT *Arabidopsis* Columbia-0 ecotype were employed. Seeds were stratified, kept at 4 °C for 3 days in the dark and then germinated and grown in hydroponic system [1] at 22 °C under controlled conditions, initially in short days (8 h light, 16 h dark) and after 21 days the seedlings were transferred to long-day (16 h light, 8 h dark) photoperiod under 100–120 mmol m^−2^ s^−1^. Plants grown under these conditions were used for determining rosette leaf number (Fig. 2A) by individual counting and rosette diameter (Fig. 2B) quantified by image analyses using the software Imagej (http://rsb.info.nih.gov). Cuantitative data were statistically analyzed with ANOVA and *T*-Student test at (*α*=0.05).

### 2.3 Massive mRNA-sequencing by Illumina, bioinformatics analysis and data processing

For RNA-Sequencing by illumina HiSeq was used total RNA from rosette leaves of 35-day old of AtFTOE 2.1 line and Wild Type (WT) plants. RNA was extracted with the RNeasy Plant Kit (Qiagen). The RNA-seq experiments were conducted with RNA isolated from three biological replicates per AtFTOE 2.1 line and two biological replicates for WT with accession numbers SRR2094583 and SRR2094587 respectively. Illumina sequencing was performed at Otogenetics (Georgia-USA). Illumina HiSeq Sequencing with PE50 yielded 20 million reads by triplicate (Table 2). The raw data files are available at the National Center for Biotechnology Information (NCBI) Sequence Read Archive (SRA) accession numbers SRR2094583 and SRR2094587 for AtFTOE replicates 1–3 and AtWT for control replicates 1–2 respectively. The paired-end reads were aligned to the reference *Arabidopsis* genome using Tophat (version 2.0.10) [7] and Bowtie2 (version 2.1.0) [4]. The reference *Arabidopsis* genome and gene model annotation files (TAIR10<ftp://igenome:G3nom3s4u@ussd-ftp.illumina.com/*Arabidopsis*_*thaliana*/NCBI/TAIR10/*Arabidopsis*_*thaliana*_NCBI_TAIR10.tar.gz>) were downloaded from the Illumina iGenomes (http://support.illumina.com/sequencing/sequencing_software/igenome.html). Differential expression was determined by Cufflinks (version 2.1.1) as described by Trapnell et al. [8] and then was visualized by CummeRbound, an edgeR package [3]. To analyze the variation in expression between two replicates from WT and three replicates from AtFTOE it was calculated the absolute difference of the log2 fold change and adjusted to *P*-value ≤0.05.

### 2.4 Visualization of differential expression by Mapman

**Fig. 3:**
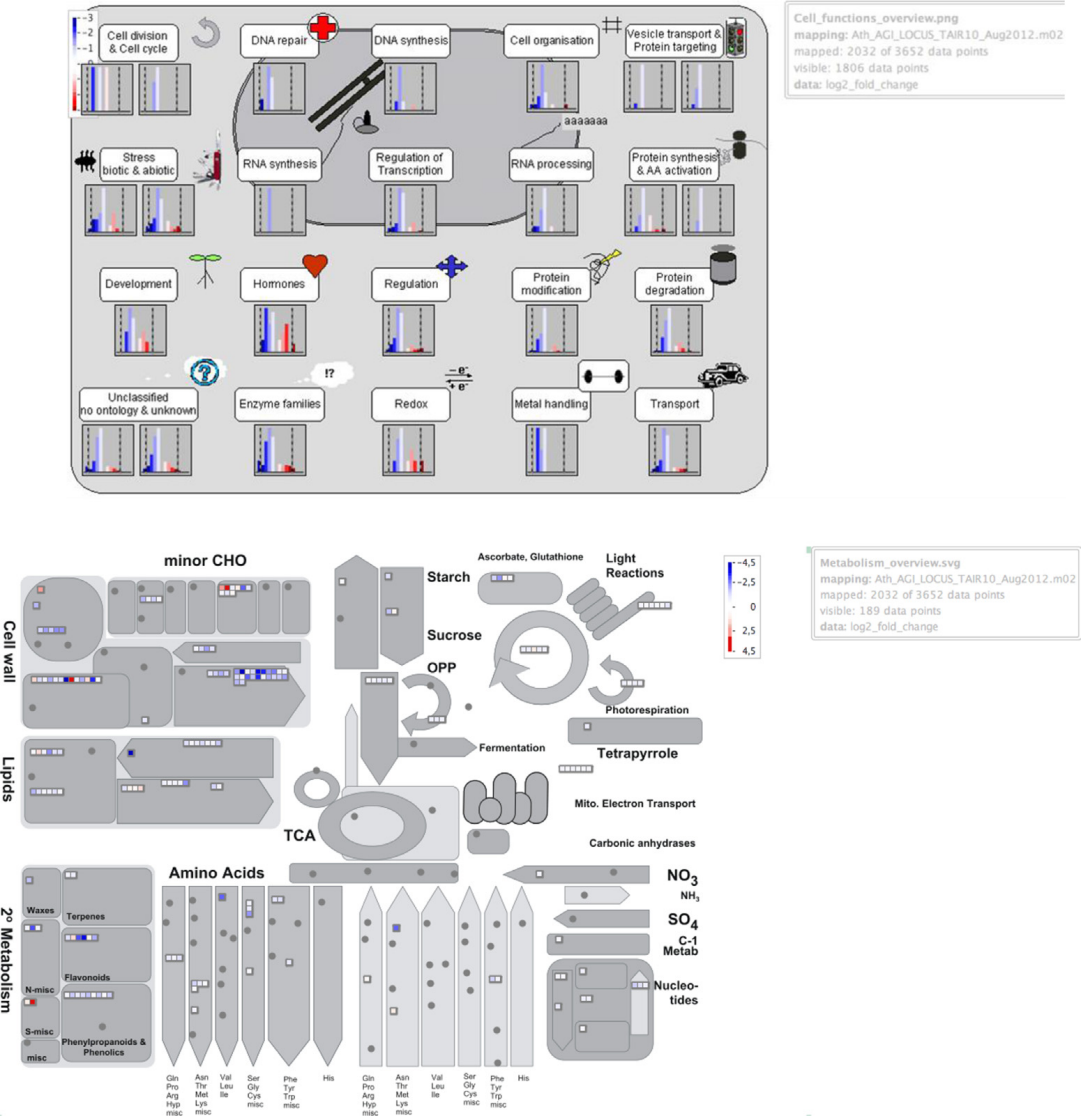
Mapman visualization showing the observed differential expression patterns, based on the Log_2_FCs of mRNA levels, in rosette leaves of three AtFTOE versus WT. Red color indicates up-regulated genes and blue color indicates down-regulated genes (A) Cell functions overview (B) Metabolism overview each gene is symbolized by a box.

In order to visualize differential expression we used Mapman tool, mapping the Mapman databases (http://mapman.gabipd.org/web/guest/mapman; Ath_AGI_LOCUS_TAIR10_Aug2012-3.m02; [6]) using raw data of differential expression log2 fold change and adjusted to *P*-value ≤0.05 (Fig. 3).

### 2.5 Primer design

Primers design (Table 1) was performed by OligoArchitect™ Primer and Probe Design Solutions (http://www.sigmaaldrich.com/technical-documents/articles/biology/probe-design-services.html). Gene sequences were obtained from Tair database (https://www.arabidopsis.org).

## Figures and Tables

**Table 1:** Primers to detect differentially expressed genes.

Gene	Locus ID	Primer Forward 5′−3′	Primer Reverse 5′−3′
AGAMOUS-LIKE 68,/MAF5	AT5g65080	MAF5- RTSYBG (F) GGAAGAAGAAGAGTAGAGAT	MAF5-RTSYBG (R) ACAGAGAATTGAGAGTTGA
Squamosa promoter-binding-like protein 4 SPL4	AT5g65080	SPL4-RTSYBG (F)GTCGGAGAGGAATCAATG	SPL4-RTSYBG (R) GGCATAGGAAGTGTCATC
MADS – BOX PROTEIN SOC1	AT2g45660	SOC1- RTSYBG (F) GAAGAGAATAGAGAATGC	SOC1-RTSYBG (R) AGAGAAGATGATAAGAGAA
ATSTP13, MSS1, STP13, SUGAR TRANSPORT PROTEIN 13	AT5G26340	AtSTP13-qPCR(F) ACTCCGTTGACAAAGTCGGT	AtSTP13-qPCR(R) TGGCGATTACGACTTGAGAG

**Table 2:** RNA sequences obtained by Illumina HighSeq2000/2500 PE100 sequencer.

**Sample**	Total bases*	**Total reads**
WT1	2,612,645,388	24,647,598
WT2	2,770,468,788	26,136,498
FT1	2,474,087,064	23,340,444
FT2	2,455,442,088	23,164,548
FT3	2,257,321,728	21,295,488

**Table 3:** List of *Arabidopsis* floral repressors downregulated in AtFTOE 2.1 line.

Genes	Locus ID	Localization	Description	FC
	At3g27200	Plasma membrane	Plastocyanin-like domain-containing protein. Cupredoxin superfamily protein.	0.11
				
SPL2, SQUAMOSA PROMOTER BINDING PROTEIN-LIKE 2	At5g43270	Nucleus	Member of the SPL (squamosa-promoter binding protein-like) gene family, a novel gene family encoding DNA binding proteins and putative transcription factors.	0.57
AP2, APETALA 2, ATAP2, FL1, FLO2, FLORAL MUTANT 2, FLOWER	AT4G36920	Nucleus	Encodes a floral homeotic gene, a member of the AP2/EREBP (ethylene responsive element binding protein) class of transcription factors and is involved in the specification of floral organ identity, establishment of floral meristem identity, suppression of floral meristem indeterminacy, and development of the ovule and seed coat. AP2 also has a role in controlling seed mass.	0.46
	At3g45160		Putative membrane lipoprotein.	0.47
				
ATMYB29	At5g07690	Nucleus	Encodes a putative transcription factor.	0.56
CA1	At3g01500	Apoplast, chloroplast stroma, thylakoid membrane, plasma membrane	Encodes a putative beta-carbonic anhydrase betaCA1.	0.44
	At3g05730	Extracellular region	Encodes a defensin-like (DEFL) family protein.	0.27
	At5g05960	Extracellular region	Bifunctional inhibitor/lipid-transfer protein/seed storage 2S albumin superfamily protein.	0.23
				
BETA CA2	At5g14740	Apoplast, chloroplast, chloroplast stroma, thylakoid membrane, cytoplasm	Encodes a beta carbonic anhydrase likely to be localized in the cytoplasm.	0.41
	At3g26960	Extracellular region	Pollen Ole e 1 allergen and extensin family protein.	0.37
				
BETA GLUCOSIDASE 33, BGLU33	At2g32860	Chloroplast	Beta glucosidase	0.27
XCP2, XYLEM CYSTEINE PEPTIDASE 2	At1g20850	Chloroplast, extracellular space, lysosome	Cysteine-type peptidase activity, peptidase activity	0.15
	At4g30650	Cell wall, chloroplast, extracellular space, lysosome	Xylem cysteine peptidase 2 (XCP2)	0.50
	At1g29660		GDSL-motif esterase/acyltransferase/lipase.Enzyme group with broad substrate specificity that may catalyze acyltransfer or hydrolase reactions with lipid and non-lipid substrates.	0.24
				
TON1 RECRUITING MOTIF 14, TRM14	At3g61380	Nucleus, plasma membrane	Phosphatidylinositol N-acetyglucosaminlytransferase subunit P-related.	0.39
ATIMD3, IMD3, IPMDH1	At1g31180	Chloroplast stroma, mitochondrion, plastid, thylakoid	The AtIMD3 is one out of 3 genes encoding the enzyme 3-isopropylmalate dehydrogenase involved in leucine biosynthesis in *Arabidopsis*. Its subcellular location has been targeted to plastids.	0.61
OEP6, OUTER ENVELOPE PROTEIN 6	At3g63160	Chloroplast, chloroplast envelope, chloroplast outer membrane, chloroplast thylakoid membrane, thylakoid	Molecular_function unknown	0.48
	At1g35190	Cytoplasm	2-oxoglutarate (2OG) and Fe(II)-dependent oxygenase superfamily protein	0.55
				
AGAMOUS-LIKE 19, AGL19, GL19	At4g22950	Nucleus	MADS-box protein AGL19.	0.23
	At3g27200		Cupredoxin superfamily protein,electron carrier activity, copper ion binding.	0.11
	At2g39850	Extracellular region	Subtilisin-like serine endopeptidase family protein; metabolic process, proteolysis.	0.56
	At3g53100	Extracellular region	GDSL-motif esterase/acyltransferase/lipase,hydrolase activity, acting on ester bonds.	0.33
	At5g44400	Cell wall, cytoplasm, plasmodesma	FAD-binding Berberine family protein.	0.11
				
MORPHOGENESIS OF ROOT HAIR 1, MRH1	At4g18640	Chloroplast	Protein serine/threonine kinase activity.	0.11
ATPAP3, PAP3, PURPLE ACID PHOSPHATASE 3	At1g14700	Extracellular region, vacuole	acid phosphatase activity, metal ion binding, protein serine/threonine phosphatase activity.	0.25
	At3g29030	Cell wall, extracellular region, membrane	Encodes an expansin. Naming convention from the Expansin Working Group.	0.17
	At1g04040	Cell wall, plant-type cell wall, plasmodesma, vacuolar membrane, vacuole	HAD superfamily, subfamily IIIB acid phosphatase	0.27
				
TPPH, TREHALOSE-6-PHOSPHATE PHOSPHATASE H	At4g39770	Cytoplasm, cytosol, nucleus	Trehalose biosynthetic process.	0.61
	At5g63180	Pectin lyase fold/virulence factor	Pectin lyase-like superfamily protein	0.35
