# Supplementary material for: Dataset of Arabidopsis plants that overexpress FT driven by a meristem-specific KNAT1 promoter
Source: Data Brief. 2016 Jun 8;8:520–8. doi: 10.1016/j.dib.2016.06.002 (PMC4919726; doi:10.1016/j.dib.2016.06.002)
Supplement: Supplementary material [file mmc1.pdf]

Conflicts of Interest Statement

Manuscript title:

Dataset of Arabidopsis plants that overexpress FT driven by a meristem-specific KNAT1 promoter

The authors whose names are listed immediately below certify that they have NO affiliations with or involvement in any organization or entity with any financial interest (such as honoraria; educational grants; participation in speakers' bureaus; membership, employment, consultancies, stock ownership, or other equity interest; and expert testimony or patent-licensing arrangements), or non-financial interest (such as personal or professional relationships, affiliations, knowledge or beliefs) in the subject matter or materials discussed in this manuscript.

Author names:

Lilian Duplat-Bermúdez
Roberto Ruiz-Medrano
David Landsman
Leonardo Mariño-Ramírez
Beatriz Xoconostle-Cázares

The authors whose names are listed immediately below report the following details of affiliation or involvement in an organization or entity with a financial or non-financial interest in the subject matter or materials discussed in this manuscript. Please specify the nature of the conflict on a separate sheet of paper if the space below is inadequate.

Author names:

This statement is signed by all the authors to indicate agreement that the above information is true and correct (*a photocopy of this form may be used if there are more than 10 authors*):

Author's name (typed)	Author's signature	Date
Lilian Duplat-Bermúdez		May 23, 2016
Roberto Ruiz-Medrano		May 24, 2016
David Landsman		May 25, 2016
Leonardo Mariño-Ramírez		May 24, 2016
Beatriz Xoconosle-Cázares		May 24, 2016
